# Aplisulfamines, New Sulfoxide-Containing Metabolites from an *Aplidium* Tunicate: Absolute Stereochemistry at Chiral Sulfur and Carbon Atoms Assigned Through an Original Combination of Spectroscopic and Computational Methods

**DOI:** 10.3390/md10010051

**Published:** 2012-01-04

**Authors:** Anna Aiello, Ernesto Fattorusso, Concetta Imperatore, Paolo Luciano, Marialuisa Menna, Rocco Vitalone

**Affiliations:** 1 The NeaNat Group, Dipartimento di Chimica delle Sostanze Naturali, Università degli Studi di Napoli "Federico II", Via D. Montesano 49, 80131 Napoli, Italy; Email: aiello@unina.it (A.A.); fattoru@unina.it (E.F.); cimperat@unina.it (C.I.); rocco.vitalone@unina.it (R.V.); 2 C.S.I.A.S. (Centro Servizi Interuniversitario di Analisi Strumentale), Università degli Studi di Napoli "Federico II", Via D. Montesano 49, 80131 Napoli, Italy; Email: pluciano@unina.it

**Keywords:** ascidian, sulfoxide, stereochemical analysis, computational methods, ECD

## Abstract

Two new sulfoxide-containing metabolites, aplisulfamines A (**1**) and B (**2**), have been isolated from an *Aplidium* sp. collected in the Bay of Naples. Their planar structure and geometry of a double bond were readily determined by using standard methods, mainly NMR spectroscopy. An original approach was used to assign the absolute configuration at the three contiguous chiral centers present in the structures of both aplisulfamines, two at carbon and one at sulfur. This involved Electronic Circular Dichroism (ECD) studies, *J*-based configuration analysis and Density Functional Theory (DFT) calculations and represents an interesting integration of modern techniques in stereoanalysis, which could contribute to the enhancement of theoretical protocols recently applied to solve stereochemical aspects in structure elucidation.

## 1. Introduction

**Figure 1:**
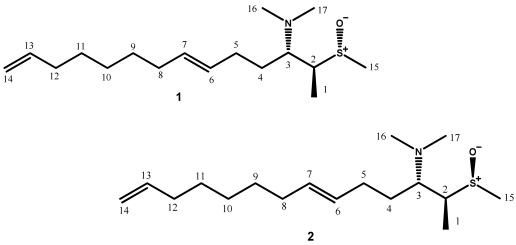
Structures of compounds **1** and **2**.

The occurrence of sulfoxides in nature is limited, being confined mostly to peptide derivatives containing methionine- or β,β-dimethylmethionine S-oxides [1,2], podolactones [3], compounds from onions and other *Allium* species [4], as well as few marine secondary metabolites [5,6,7,8]. As a part of our ongoing search for new and/or bioactive metabolites from Mediterranean ascidians, two novel natural sulfoxides, aplisulfamines A (**1**) and B (**2**), have been isolated from the tunicate *Aplidium* sp. collected in the Bay of Naples. We here report the isolation and structure determination of these two unique compounds (Figure 1). Particularly, an original approach was used to solve the absolute configuration at the three contiguous chiral centers of the molecules, two at carbon and one at sulfur. This represented an interesting integration of modern techniques in stereoanalysis and involved ECD studies, J-based configuration analysis and Density Functional Theory (DFT) calculations. Aplisulfamines A and B were tested for their cytotoxic and/or antiproliferative effects on cancer cells as well as for their antimicrobic properties; in both cases, the new metabolites did not show significant activity.

## 2. Results and Discussion

Specimens of *Aplidium* sp. (150 g dry wt.) collected at Pozzuoli (Naples, Italy) were homogenized and extracted with MeOH. The concentrated extract was partitioned between H_2_O and EtOAc and the organic layer was dried and then subjected to silica gel MPLC (hexane→EtOAc→MeOH). Fractions eluted with EtOAc/MeOH 9:1 were separated and purified by repeated RP-HPLC’s to give **1** (9.0 mg) (*t*_R_ = 11.7 min) and **2** (3.0 mg) (*t*_R_ = 13.9 min).

HRESI mass spectrum (positive ions) of **1** revealed two pseudomolecular ion peaks at *m/z* 300.2351 and 322.2171 corresponding to [M + H]^+^ (calculated value: 300.2356) and [M + Na]^+^ (calculated value: 322.2175), respectively. The molecular formula C_17_H_33_NOS was thus established for **1** which corresponded to two unsaturation degrees. The ^13^C NMR spectrum (CD_3_OD) evidenced the presence of 4 olefin carbons (3 methines and 1 methylene), which accounted for the two unsaturation degrees implied by the molecular formula. Therefore, **1** must be acyclic.

^1^H-NMR spectrum exhibited a number of well resolved signals, which were associated to the bonding carbons by analysis of HSQC spectrum (Table 1).

Inspection of COSY and TOCSY spectra revealed that most of the proton signals belong to a single spin system, corresponding to the whole linear tetradeca-6,13-diene skeleton of **1**. The *trans* geometry of C6 double bond was indicated by the large coupling constant between H6 and H7 as well as by the ^13^C chemical shift of C5 and C8 allylic carbons (Table 1) [9,10,11]. In addition to signals of the above spin system, NMR spectra showed signals indicative of the presence of three methyl groups. Chemical shift values (δ_H_ 2.43; δ_C_ 30.4) of one of them were consistent with those of a methyl sulfoxide group; this agreed with a facile loss of 64 amu observed in the ESI MS/MS spectrum of **1**, attributable to expulsion of CH_3_SOH from the pseudomolecular ion at *m/z* 300.12. HMBC correlations were observed between the protons of this methyl group (Me-15) and C2 (δ 58.2), as well as between the methine proton signal at δ 3.31(H2) and C15; this allowed to locate the methyl sulfoxide functionality at C2. The remaining two methyls were assigned to those of an *N*,*N*-dimethylamino group (δ_H_ 2.29, 6H, s; δ_C_ 41.0) linked at C3; this was consistent with the long-range coupling observed between the N-flanking methyl protons and C3.

The most challenging task in the structure elucidation of aplisulfamine A (**1**) was the assignment of its configurational pattern, since **1** contains three adjacent stereocenters in a conformationally flexible system. This sterochemical problem was solved by combination of NMR and ECD experiments assisted by computational studies through a three stage-approach involving the sequential assignment of: (i) sulfur absolute configuration; (ii) relative configuration of the C2-C3 segment; (iii) relative configuration of the S-C2 segment.

The assignment of the absolute configuration at sulfur was achieved by ECD studies. Mislow *et al*. have shown that a correlation exists between the absolute configuration of methyl alkyl sulfoxides and their optical activity in terms of the signs and rotational strengths of appropriate Cotton effects [12]. In the absence of strongly perturbing groups, as in the case of alkyl allyl or diallyl sulfoxides [13], a negative Cotton effect, centered at the absorption band near in the region 220–230 nm in acetonitrile, correlates with the ***R*** configuration. This rule was found to still be applicable when the alkyl group itself is also chiral [14] as in the case of aplisulfamine A (**1**). 

**Figure 2:**
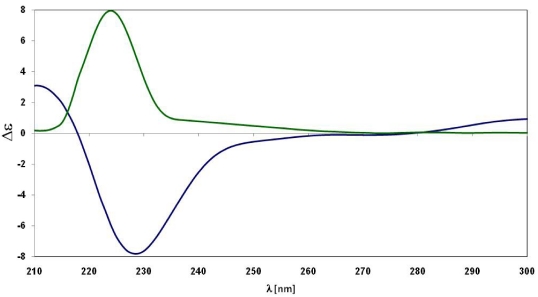
ECD curves of compounds **1** (blue) and **2** (green).

Therefore, by application of Mislow’s rule [12], the *R* configuration could be assigned to the sulfur atom in **1** because of the negative sign of the Cotton effect observed in its ECD spectrum (Figure 2).

The relative configuration of C2-C3-segment of **1** was then assigned through a *J*-based NMR configurational analysis according to the method proposed by Murata and coworkers [15]. This method has proved particularly useful for the stereochemical analysis of acyclic structures with adjacent stereogenic carbons bearing hydroxy, alkoxy or ethyl substituents and, successively, it has been extended to chlorine- and nitrogen-containing systems [16,17,18].

**Figure 3:**
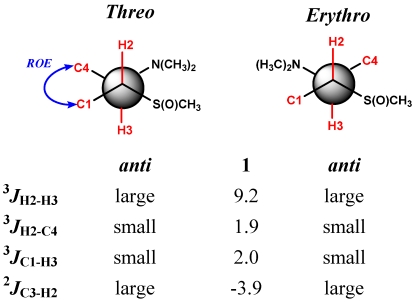
^3^*J*_H-H_ and ^2,3^*J*_C-H_ (Hz) values of C2-C3 segment of **1**.

We could now extend its application to a sulfoxide-substituted chain, due to the existence of a staggered dominant conformation in C2-C3 segments as evidenced by the large ^3^*J*_H2-H3_ value (9.2 Hz). Evaluation of the complete set of homo- and hetero-nuclear *J* values pointed to the two rotamers with an H/H anti orientation among the six staggered rotamers of the two possible relative configurations (threo and erythro) of the stereopair (Figure 3).

In this case the unassisted knowledge of coupling constants does not allow discrimination between the two conformers with opposite relative configuration and additional spatial information, typically dipolar effects, is required [15]. A strong correlation between protons on C1 and C4 was observed in the ROESY spectrum of **1**, indicative of a gauche orientation for Me-1 and CH_2_-4 (Figure 3). This dipolar coupling allowed the threo stereochemical relationship (2*S*, 3*S* or 2*R*, 3*R*) for the pair of vicinal asymmetric carbons C2 and C3 to be assigned. Two stereoisomers of **1** met with the whole results so far reported, (2*S*,3*S*,*E*)- and (2*R*,3*R*,*E*)-*N*,*N*-dimethyl-2-[(*R*)-methylsulfinyl]tetradeca-6,13-dien-3-amine.

**Figure 4:**
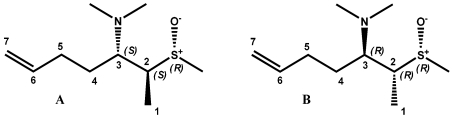
Models A and B.

The assignment of the relative configuration of S-C2 segment allowed the right selection between the two alternatives. For this task we started from the analysis of ROESY spectra; strong dipolar couplings of Me-15 with Me-1 and H3 were observed in **1**, while no ROE correlation was present with H2. This suggested the presence, around the S-C2 bond, of a preferential rotamer in which Me-15 is gauche to Me-1 and H3, and anti to H2. This information turned out a key datum in the light of the results of a conformational analysis carried out on models A and B (Figure 4), which paralleled the functionalized part of the two alternative stereoisomers of **1**. 

Through a DFT (Density Functional Theory) approach we have identified a series of conformers resulting from rotation around the S(O)-C2 bond in models A and B. 

In order to generate a rational number of conformations for this highly flexible substance, the torsion angles C2-C3 have been fixed on the basis of the size of ^3^*J*_H2-H3_ coupling constant and the conformational explorations for the O-S-C2-C1 dihedral angles were achieved within a dihedral angle range from +48° to −48°. This systematic search afforded 12 rotamers around S-C2 bond for each stereoisomeric structures A and B; they were geometrically optimized at DFT level using a B3LYP functional and 6-31G(d) basis set. Then, the relative energies of all conformations were calculated with respect to the dihedral angle O-S-C2-C1 and equilibrium room-temperature Boltzmann populations obtained thence. All DFT calculations were carried out using the GAUSSIAN 03 program.

**Figure 5:**
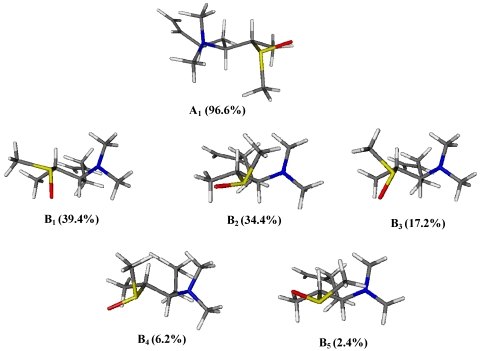
Dominant rotamers of models A and B with the relevant Boltzmann populations.

**Figure 6:**
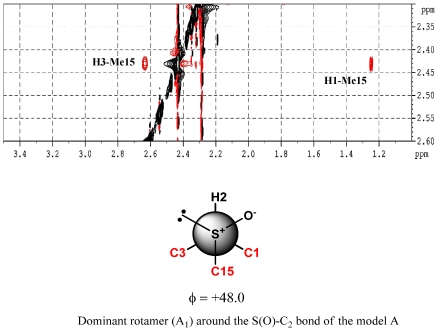
Key ROE correlations of compound **1**.

As shown in Figure 5, structure A is characterized by a sole dominant rotamer (96.6% of total population) while, for structure B, five of the 12 rotamers have energies within 3 kcal/mol of the lowest energy conformation and are significantly populated (39.4%, 34.4%, 17.2%, 6.2%, and 2.4%). However, only the lowest energy conformer of model A meets with ROESY spatial information obtained for **1.** In this structure, Me-15 is gauche to both Me-1 and H3 while it is anti to H2 (Figure 6). Notwithstanding the appearance of the Newman model of rotamer A1, actually in this conformation the steric interactions between C15, C3, and C1 are probably reduced due to the features of the S-C bonds (C15-S and C2-S). They are indeed longer (≈1.8 Å) when compared to C-C bonds (≈1.5 Å), lowing the torsional barrier that would normally be expected. So, relative configuration of S-C2 segment in **1** was believed to be the same as model A, suggesting the conclusive stereostructure of aplisulfamine A to be (2*S*,3*S*,*E*)-*N*,*N*-dimethyl-2-[(*R*)-methylsulfinyl]tetradeca-6,13-dien-3-amine. 

Quantum-mechanical prediction of both ^13^C NMR chemical shifts and ECD spectra of models A and B strongly corroborated the proposed stereochemistry. Briefly (more details are reported in Supporting Information), ^13^C NMR chemical shifts of the lowest energies conformers of the models A and B were calculated, using the MPW1PW91 functional and the 6-31G(d, p) basis set. 

Chemical shift values for structure B were obtained considering the Boltzmann weighted GIAO (Gauge Including Atomic Orbitals) average derived from the energies of single conformers. The calculated values were compared to the experimental ones relevant to the C1/C5 segment of 1. Mean absolute error (MAE) values obtained for each stereoisomer indicated the best fit with the experimental data relevant to the C1/C5 segment of 1 for model A (1.40 *versus* 3.43 of model B) [19].

**Figure 7:**
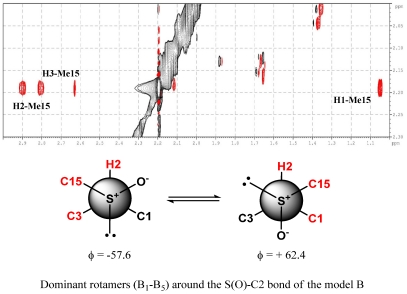
Key ROE correlations of compound **2**.

The ECD spectra of model A and model B were also simulated. The excitation energies as well as the oscillator and rotatory strengths of the electronic excitation were calculated for the lowest energies conformational families of each structure using the TDDFT methodology [20]. The theoretical curves of models A and B *versus* the experimental one of aplisulfamine A are reported in Figure 7; their courses, all with a negative sign, strongly supported the stereochemistry assignment at sulfur in **1**.

Spectral data obtained for compound **2** were nearly identical to those of **1**; the ESI mass spectrum (positive ion mode) of aplisulfamine B (**2**) exhibited the same pseudomolecular and fragment ions present in the spectrum of **1**, thus indicating that the two compounds were isomers with the molecular formula of C_17_H_33_NOS. Comparison of their ^13^C and ^1^H NMR spectra showed few differences, the only major being associated with the resonances relevant to the most functionalized part of the molecules (see Table 1). 

Analysis of 2D NMR data of **2** revealed it possessed the same *N*,*N*-dimethyl-2-[methylsulfinyl]tetradeca-6,13-dien-3-amine planar structure of **1**; the *trans* geometry was also assigned to C6 double bond on the basis of *J*_H6-H7_ value (15.1 Hz) and the chemical shifts of C5 and C8 allylic carbons. This information led to the conclusion that **1** and **2** were diastereoisomers differing in the absolute configuration of one or two of the chiral atoms.

The assignment of absolute stereochemistry of **2** was achieved following the same procedure as described for **1**. On the basis of the positive sign of the Cotton effect in the ECD curve (Figure 2), the *S* absolute configuration was assigned to the sulfur atom in **2**, opposite to that in **1**. Furthermore, the *threo* relative stereochemistry was assigned to C2-C3 segment through evaluation of the homo- and hetero-nuclear *J* values and ROESY data of **2** (see Supporting Information).

**Figure 8:**
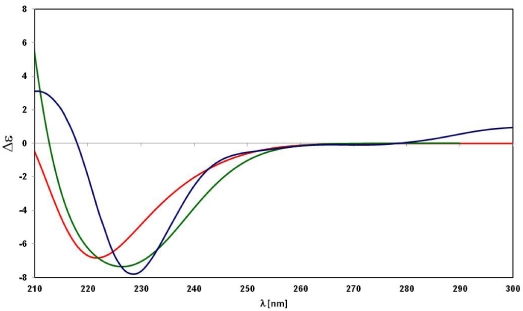
Theoretical ECD curves of models A (green) and B (red) *vs.* experimental ECD curve of aplisulfamine A (blue).

The results obtained from these first two stages of the configurational analysis of **2** already led to establish its absolute stereochemistry to be (2*S*, 3*S*, S*S*), discarding the alternative (2*R*, 3*R*, S*S*) configuration since it was enantiomeric to that of **1**. Moreover, conformational analysis performed on models A and B (Figure 5) as well as DFT calculations of their ^13^C NMR chemical shifts strongly supported the proposed stereochemistry. Indeed, the finding of **2** constituted good evidence that arguments on which we have based the relative configuration of S-C2 segment assignment in **1** were sound. In fact, as expected, the ROESY spectrum of **2** showed correlations between Me-15 (δ 2.20)and Me-1, H2 and H3 (Figure 8), according with the structure of the lowest energy rotamers of model B (Figure 4) which is enantiomeric to the configuration proposed for **2**. Furthermore, comparison of theoretical ^13^C NMR chemical shifts of models A and B with the experimental ones of the C1/C5 fragment of **2** gave a MAE value of 2.72 for model B *versus* 3.91 for model A. 

**Table 1:** ^1^H and ^13^C NMR (CD_3_OD) of **1** and **2**.

POS	1		2	
	δ_C_^a^	δ_H_ (mult, *J* in Hz)^b^	δ_C_^a^	δ_H_ (mult, *J* in Hz)^b^
**1**	7.8	1.25 (d, 6.8 )	9.4	1.06 (d, 6.6 )
**2**	58.2	3.31(dq, 9.2,6.8)	60.0	2.91(dq, 10.6,6.6)
**3**	65.2	2.64(m)	65.2	2.81 (dt, 10.6,4.4)
**4a**	28.8	1.43^c^	25.2	1.70 ^c^
**4b**		1.78 (m)		1.90 (m)
**5**	32.5	2.12(m)	31.2	2.17 (m)
**6**	130.6	5.42(bdt 15.2, 6.2)	132.7	5.41(bdt 15.1, 6.5)
**7**	132.4	5.47(bdt 15.1, 6.2)	132.7	5.47(bdt 15.1, 6.5)
**8**	33.5	2.00(m)	34.6	2.02 (m)
**9**	30.5	1.38 ^c^	30.0	1.40 ^c^
**10**	29.7	1.33 ^c^	30.0	1.40 ^c^
**11**	30.0	1.39 ^c^	30.0	1.40 ^c^
**12**	34.9	2.05(q 6.7)	31.1	2.04 (m)
**13**	140.0	5.80(ddt, 17.1, 10.2, 6.7)	138.8	5.80(ddt, 17.1, 10.2, 6.7)
**14a**	114.7	4.92 (bdd, 10.2, 2.0)	114.7	4.92 ^c^
**14b**		4.98 (bdd, 17.1, 2.0)		4.98(bdd, 17.1, 2.0)
**15**	30.4	2.43 (s)	40.0	2.20 (s)
**16/17**	41.0	2.29 (s)	38.0	2.63 (s)

^a^ Spectrum recorded in CD_3_OD at 125 MHz. ^b^ Spectrum recorded in CD_3_OD at 500 MHz. ^c^ Partially overlapped by other signals.

## 3. Experimental Section

### 3.1. General Experimental Procedures

ESI mass spectra were recorded on a hybrid quadrupole-TOF mass spectrometer in MeOH. The spectra were recorded by infusion into the ESI source using MeOH as the solvent. HRESIMS (positive mode) were performed on a Thermo LTQ Orbitrap XL mass spectrometer. Optical rotations were measured on a Jasco DIP 370 digital polarimeter. at 589 using a 10-cm microcell. ^1^H (700 MHz and 500 MHz) and ^13^C (175 MHz and 125 MHz) NMR spectra were recorded on a Varian INOVA spectrometer respectively; chemical shifts were referenced to the residual solvent signal (CD3OD: δH = 3.31, δC = 49.0). For an accurate measurement of the coupling constants, the one-dimensional ^1^H NMR spectra were transformed at 64 K points (digital resolution: 0.09 Hz). Homonuclear ^1^H connectivities were determined by COSY and TOCSY (mixing time 100 ms) experiments. Through-space ^1^H connectivities were evidenced using a ROESY experiment with a mixing time of 500 ms. Two and three bond ^1^H-^13^C connectivities were determined by gradient 2D HMBC experiments optimized for a ^2,3^*J* of 8 Hz. ^3^*J*_H-H_ values were extracted from 1D ^1^H NMR and 2D E.COSY spectra. For the E.COSY spectrum 32 scans per t1 value were acquired with a t1max of 65 ms. ^2,3^*J*_C-H_ values were obtained from phase-sensitive PFG-PS-HMBC spectra and ^13^C coupled and decoupled HSQC-TOCSY according the following conditions. The PFG-PS-HMBC spectrum was recorded using 2K points in ω2, setting the delay for long-range coupling evolution (Δ) at 50 ms, with 32 scans/t1 (t1max 15.2 ms). Zero-filling (8 K × 1 K) was carried out in ω2 and ω1, respectively, to obtain a digital resolution of 0.9 Hz in ω2. The ^13^C coupled and decoupled HSQC-TOCSY were recorded using 2K points in ω2, setting the mixing time 80 ms and ^1^*J*_CH_ of 140 Hz, with 120 scans/t1 (t1max 9.7 ms). Zero-filling (8 K × 1 K) was carried out in ω2 and ω1, respectively, to obtain a digital resolution of 0.5 Hz in ω2; the proton-carbon ^2,3^*J* coupling were extracted trough a computer-aided analysis of the heteronuclear coupled and decoupled multiplets acquired in two separate experiments, according to the method proposed by Kover *et al.* [21]. All spectra were acquired at 278^o^ K and the samples were prepared by dissolving compounds 1 and 2 in 0.5 mL of CD_3_OD (Armar, 100% D). 

### 3.2. Collection, Extraction and Isolation

Specimens of *Aplidium* sp. were collected at Pozzuoli (Naples, Italy), frozen immediately and kept frozen until extraction. A reference specimen is deposited at the Dipartimento di Chimica delle Sostanze Naturali, University of Naples “Federico II”. Fresh thawed animals (8.5 g dry weight after extraction) were homogenized and extracted twice with methanol and then twice with chloroform (4 × 200 mL). The combined extracts were concentrated *in vacuo* and the resulting aqueous residue was extracted with EtOAc. Separation of the EtOAc soluble material (480 mg) was achieved by gradient silica gel MPLC (hexane→EtOAc →MeOH). Fractions eluted with EtOAc/MeOH 9:1 (v/v) were combined and the solvent was evaporated yielding a residue (80 mg) which was subjected to a first chromatography by HPLC on a C18 column (Luna 5 μm, 250 × 3.00 mm) eluting with MeOH/H_2_O 85:15 (v/v). Final purification was achieved by a further HPLC separation on a chiral column (Chirex 5001 5 μm, 100 × 4.60 mm) in the same elution conditions, which afforded aplisulfamine A (9.0 mg, 0.10% of dry weight) and aplisulfamine B (3.0 mg, 0.03% of dry weight) in the pure state. 

Aplisulfamine A (1). [α]D = +15.6 (methanol, c = 0.001); HRESI-MS (positive ion mode): m/z (%) = 300.2351 [M + H]^+^, 322.2171 [M + Na]^+^; ^1^H and ^13^C NMR data (CD_3_OD) are reported in Table 1.

Aplisulfamine B (1). [α]_D_ = −9.7 (methanol, c = 0.001); HRESI-MS (positive ion mode): m/z (%) = 300.2349 [M + H]^+^, 322.2168 [M + Na]^+^; ^1^H and ^13^C NMR data (CD_3_OD) are reported in Table 1.

### 3.3. Computational Details

DFT calculations were performed on a Pentium-4 processor at 3.0 GHz using the Gaussian03 package (Multiprocessor). Systematic Conformational Search for the models A and B around S-C2 bond was carried out at the B3LYP level using the 6-31G(d) basis set (range: +48 to −48; step: 30^o^; number of conformers = 12). All the conformers obtained were subsequently optimized at the B3LYP level using the 6-31G(d,p) basis set. 

GIAO ^13^C calculations were performed using the mPW1PW91 functional and 6-31G(d,p) basis set, using as input the geometry previously optimized at the mPW1PW91/6-31G(d) level. For these calculations, the IEF-PCM solvent continum model, as implemented in Gaussian (methanol solvent), was used. TDDFT calculations were run using the functional B3LYP and the basis sets TZVP including at least 30 excited states in all cases, and using IEF-PCM for MeOH. ECD spectra were generated. 

## 4. Conclusions

The interesting combination of spectroscopic and computational methods used to assign the absolute stereochemistry of aplisulfamines and, particularly, the original approach we used to assign the relative configuration of an acyclic system composed by an asymmetric carbon linked to a chiral sulfoxide, contribute to the enhancement of theoretical protocols recently applied to solve stereochemical aspects in structure elucidation.

The isolation of both epimers at sulfur brings into question whether they are both of natural origin or, instead, the natural product is only one of them which underwent racemization at the sulfur site during the extraction or partition steps. The latter hypothesis cannot be excluded; actually, the isolation of both aplisulfamines A and B was indeed very fortunate. Having both epimers at the chiral sulfoxide available, we could indeed make a strong case for the stereochemistry assignment at sulfur by application of Mislow’s rule as well as for the conformational analysis of S-C2 segment which succeeded in assigning C2 absolute stereochemistry. In addition, the ECD spectra of the natural aplisulfamines, along with the theoretical curves of Models A and B obtained from DFT calculations, are further examples which could implement the case study of Cotton effects of substituted sulfoxides. Our studies evidenced that also for aplisulfamines, containing both a heteroatomic substituent and a chiral secondary carbon directly linked at the sulfoxide group, a simple correlation exists between the sign of their Cotton effects and the sulfur atom configuration.
